# Anti-biofilm effect**s of anthranilate on a broad range of bacteria**

**DOI:** 10.1038/s41598-017-06540-1

**Published:** 2017-08-17

**Authors:** Xi-Hui Li, Soo-Kyoung Kim, Joon-Hee Lee

**Affiliations:** Department of Pharmacy, College of Pharmacy, Pusan National University, Busan, 609-735 South Korea

## Abstract

Anthranilate, one of tryptophan degradation products has been reported to interfere with biofilm formation by *Pseudomonas aeruginosa*. Here, we investigated the effects of anthranilate on biofilm formation by various bacteria and the mechanisms responsible. Anthranilate commonly inhibited biofilm formation by *P. aeruginosa*, *Vibrio vulnificus*, *Bacillus subtilis*, *Salmonella enterica* serovar Typhimurium, and *Staphylococcus aureus*, and disrupted biofilms preformed by these bacteria. Because anthranilate reduced intracellular c-di-GMP and enhanced swimming and swarming motilities in *P. aeruginosa*, *V. vulnificus*, *B. subtilis*, and *S. enterica*, it is likely that anthranilate disrupts biofilms by inducing the dispersion of these bacteria. On the other hand, in *S. aureus*, a non-flagellate bacterium that has no c-di-GMP signaling, anthranilate probably inhibits biofilm formation by reducing slime production. These results suggest that anthranilate has multiple ways for biofilm inhibition. Furthermore, because of its good biofilm inhibitory effects and lack of cytotoxicity to human cells even at high concentration, anthranilate appears to be a promising agent for inhibiting biofilm formation by a broad range of bacteria.

## Introduction

Biofilms are surface-attached microbial communities encapsulated in extracellular polymeric substances (EPS)^1, 2^. Biofilms cause serious infections and help make infections chronic. One study showed biofilms were detected in 60% of chronic wound specimens, and according to researchers at the Centers for Disease Control and Prevention (CDC), nearly 80% of chronic infections are mediated by the microbial biofilms^3, 4^. Moreover, bacterial cells within biofilms are nearly 1000 times more resistant to antibiotics than their planktonic counterparts^5, 6^.

Many biofilm inhibitors, such as anti-microbial peptides, metal chelators, quorum sensing inhibitors, and amino acids have been discovered for decades^4, 7–11^. The inhibition of biofilm formation by D-amino acids has been reported in the gram-positive species, *Bacillus subtilis* and *Staphylococcus aureus* ^12, 13^. D-Tryptophan inhibits *B. subtilis* biofilm formation by incorporated into peptidoglycan synthesis and decreasing the expressions of the matrix-producing operons, *epsA* and *tapA* ^14^. Tryptophan has been reported to inhibit biofilm formation by the gram-negative pathogens *Escherichia coli* and *Pseudomonas aeruginosa* ^15, 16^. Furthermore, tryptophan-coated biological dressing has been reported to have a beneficial effect on wound healing and closure^17–19^.

Indole is a tryptophan metabolite and is produced by a wide range of bacterial species that harbor *tnaA* gene encoding tryptophanase^20^. In *E. coli*, indole has been shown to reduce biofilm formation by interacting with SidA-mediated transcription^21^. Its derivatives, 5-hydroxyindole and 7-hydroxyindole, have also been shown to reduce biofilm formation by *E.coli* ^22^. In recent studies, indole was found to enhance biofilm formation by *P. aeruginosa* in a quorum sensing (QS)-independent manner^21, 23^.

Some other bacteria, such as, *P. aeruginosa*, metabolize tryptophan to anthranilate through a kynurenine pathway using *kynBAU* genes^24^. Recently, it was reported in *P. aeruginosa* that anthranilate degradation is related to biofilm formation. *P. aeruginosa* mutants unable to degrade anthranilate formed less biofilm, and the expressions of genes in the anthranilate degradation pathway were enhanced during biofilm formation without activation of the synthetic pathway for *Pseudomonas* quinolone signal (PQS; 2-heptyl-3-hydroxy-4-quinolone). Since anthranilate is a precursor of PQS, this result suggests anthranilate is exhausted by degradation via the TCA cycle when biofilms are formed^25^. Later, it was found that anthranilate can disrupt biofilm structures and induce the detachment of preformed *P. aeruginosa* biofilms by reducing intracellular c-di-GMP levels and modulating the expressions of genes involved in EPS production^23^.

However, it remains unclear whether anthranilate affects biofilm formation by other microorganisms, despite its potent biofilm-inhibiting effects. In this study, we investigated the effects of anthranilate on biofilm formation by various bacteria, that is, *P. aeruginosa*, *Vibrio vulnificus*, *B. subtilis*, *S. aureus*, and *Salmonella enterica* serovar Typhimurium. We found that anthranilate disrupted biofilm formation by a wide range of bacteria and that it did so via multiple mechanisms. Since we confirmed that anthranilate has no cytotoxicity to human cells even at high concentration, we suggest that anthranilate is a promising anti-biofilm agent to inhibit biofilm formation of broad range of bacteria.

## Materials and Methods

### Bacterial strains and culture conditions

The bacterial strains and plasmids used in this study are listed in Table S1. *P. aeruginosa*, *S. aureus, B. subtilis*, and *S. enterica* strains were grown in Luria-Bertani (LB) medium at 37 °C with constant shaking (170 rpm), but the NaCl concentration was increased to 2.0% (wt/vol) for *V. vulnificus* (this medium is indicated as LBS). Anthranilate was dissolved at 1 M in dimethyl sulfoxide (DMSO) as a stock solution and diluted into media to a final concentration of 0.1 mM. Cell growth was determined by measuring optical density at 600 nm (OD_600_). To select plasmid-harboring strains, carbenicillin (Car) or erythromycin (Erm) were used at 150 μg/ml for *P. aeruginosa* strains harboring pSKcdrA or at 10 μg/ml for *S. aureus* AH1121, respectively. To induce GFP (green fluorescence protein) in *S. aureus* AH1121 strain, anhydrotetracycline (aTet) was added at 0.4 μg/ml.

### Biofilm formation and assay

Biofilm analyses were performed in the following media; modified M63 minimal medium (15 mM (NH_4_)_2_SO_4_, 100 mM KH_2_PO_4_, 1.8 μM FeSO_4_·7H_2_O, 0.2% glycerol, and 1 mM MgSO_4_) for *P. aeruginosa* ^16^, modified VFMG-CF (50 mM Tris-HCl, pH 7.2, 50 mM MgSO_4_, 300 mM NaCl, 10 mM KCl, 0.33 mM K_2_HPO_4_, 18.5 mM NH_4_Cl, and 32.6 mM glycerol, 10 mM CaCl_2_) for *V. vulnificus* ^26^, TSB medium (30 g/l of Bacto^TM^ Tryptic Soy Broth, BD) supplemented with 0.5% glucose for *S. aureus* and *B. subtilis*, and LB without NaCl but supplemented with 2% glucose for *S. enterica* ^27^. For flow cell biofilm analyses, differently diluted TSBs were used for *S. aureus*, *S. enterica* (0.6 g/l supplemented with 0.2% glucose) and *B. subtilis* (3 g/l)^28^.

The static biofilm assay was performed as previously described^16, 26^. Briefly, overnight cultures of bacterial strains were diluted 1:100 (vol/vol) in fresh medium, put into individual wells (200 μl) in 96-well polystyrene plates, and incubated for 12–16 hours at 37 °C without shaking. After measuring OD_600_, planktonic cells were poured out, and plates were washed with water and dried for 10 minutes. Then, 200 μl of crystal violet (0.1%, wt/vol) was added to each well and incubated for 10 minutes to stain biofilms attached to well surfaces. After a brief wash, the biofilm-staining crystal violet was dissolved in 200 μl of 30% acetic acid, and staining levels were assessed by measuring absorbance at 595 nm (A_595_). The data were normalized by cell growth (OD_600_). For biofilm dispersion assays, biofilms were formed in this static system for 12 hours without anthranilate. Spent medium was then discarded and planktonic cells were completely removed by washing 3 times with distilled water. Wells were then filled with 200 μl of fresh PBS containing 100 μM anthranilate. After incubation at 37 °C for 12 hours, wells were washed with distilled water, and remaining biofilms were stained with crystal violet and quantitated as described above.

Flow cell biofilm analysis was carried out as previously described^23, 26, 28, 29^. Briefly, overnight cultures of bacteria were diluted to OD_600_ of 0.01. 300 μl of the diluted cultures was then injected into each flow cell chamber, incubated for 1 hour without flow to allow attachment, and then fresh medium was pumped through the chamber using a peristaltic pump. Biofilms in flow cells were grown for 3–5 days at room temperature under constant flow, and biofilms in flow cells were visualized using GFP or by staining with 0.1% SYTO 9 for 15 minutes and analyzed by fluorescence microscopy, as described below. For the biofilm dispersion analysis, biofilms were allowed to form in a flow cell system for 2–3 days without anthranilate, and then these preformed biofilms were then treated with 0.1 mM anthranilate for 24 hours.

### Biofilm imaging and quantification

Biofilms in flow cells were imaged by confocal laser scanning microscopy (CLSM) (Olympus; FV10i) or by fluorescence microscopy (Zeiss; Axioskop FL). Three-dimensional images of biofilms were reconstructed from plane images using Bitplane Imaris 6.3.1 image analysis software. Fluorescence intensities of flow cell biofilms were quantified using ImageJ software.

### β-Galactosidase activity assay

β-Galactosidase activity was measured using the Galacto-Light Plus™ kit (Applied Biosystems) as previously described^30^. Briefly, OD_600_ values were measured and then 100 μl aliquots of cultures were mixed with 10 μl of chloroform. After vigorous vortexing and incubation for 15 minutes at room temperature, 10 μl aliquot of supernatants were transferred to new tubes and substrate solution of the Galacto-Light Plus™ kit was added. After 50-minute incubation in the dark for 50 minutes at room temperature, 150 μl of light emission solution (Accelerator II) of the kit was added and luminescence was promptly measured using a multi-well plate reader (Tristar LB941; Berthold). Measured luminescence was normalized by OD_600_ of culture and β-galactosidase activities are presented as luminescence/OD_600_.

### Motility assay

Swimming, swarming, twitching (for *P. aeruginosa*, *V. vulnificus*, *B. subtilis*, and *S. enterica*), and spreading motilities (for *S. aureus*) were measured as previously described^31–36^. Swimming was assayed on the following media; LB and LBS media solidified with 0.3% (wt/vol) agar were used for *P. aeruginosa* and *V. vulnificus*, respectively. Nutrient broth (NB) supplemented with 0.5% glucose and solidified with 0.3% agar was used for *S. enterica*, and tryptone medium (1% tryptone, 0.5% NaCl) solidified with 0.3% agar was used for *B. subtilis*. Overnight cultures (3 μl) were inoculated into the middle of agar and incubated at 37 °C for 24 hours. Degrees of swimming motility are presented as the average of the largest and smallest diameters of swimming areas. Swarming was assayed on NB medium supplemented with 0.5% glucose and solidified with 0.5% agar for *P. aeruginosa*, *B. subtilis*, and *S. enterica*, or on Brain Heart Infusion (BHI) medium containing 2% NaCl and solidified with 0.3% agar for the *V. vulnificus*. To assess swarming motilities, 3 μl of overnight culture was dropped on the surfaces of agar plates and incubated at 37 °C for 24 hours. Degrees of swarming motility are presented in the same manner as swimming motility. Twitching was assayed on LB or LBS (for *V. vulnificus*) medium solidified with 1% agar. The strains were stab-inoculated with a sharp toothpick to the bottoms of plates and incubated for 24 hours at 37 °C. Twitching zones at plate-agar interfaces were visualized by staining with 1% crystal violet and their sizes were measured as described above. Spreading motility (for *S. aureus*) was assayed on tryptone medium solidified with 0.3% agar. Briefly, 5 μl of overnight cultures were dropped onto the surfaces of agar plates and incubated at 37 °C for 24 hours. Degrees of spreading were measured as described for swimming motility.

### Measurement of intracellular c-di-GMP

Intracellular c-di-GMP levels were measured as described elsewhere^37, 38^. Bacterial cells were cultured in LB or LBS media containing 100 mM anthranilate at 37 °C with agitation for 8 hours. Cells were harvested and lysed by sonication in a buffer (10 mM Tris-HCl, 100 mM NaCl, pH 8.0). Protein concentrations of lysates were determined using Bio-Rad Protein Assay Dye Reagent (Bio-Rad). Cellular macromolecules were precipitated by adding perchloric acid (final concentration, 12%) on ice for 10 minutes, and then neutralized by adding a neutralizing buffer (3 M KOH, 0.4 M Tris, 2 M NaCl). After centrifugation, the soluble fraction was filtered through a 0.2 μm filter and 3 kD exclusion column. Thiazole orange (TO) was then added (final concentration, 30 μM) and incubated at 4 °C for 12 hours. Fluorescence was measured using a Tristar LB941 unit (Berthold; excitation 510 nm and emission 580 nm). Results were validated by comparing with independent measurements of intracellular c-di-GMP using *cdrA-lacZ* reporter in *P. aeruginosa*, as previously described^23^.

### ***In vitro*** cytotoxicity

Cytotoxicity of anthranilate was measured using the human HepG2 hepatocyte cell line, as previously described^39^. Cells were cultured in high glucose Dulbecco’s Modified Eagle Medium (DMEM, Nissui) supplemented with 100 mg/ml streptomycin, 2.5 μg/ml amphotericin B, and 10% heat-treated fetal bovine serum (FBS) at 37 °C in a 5% CO_2_ humidified incubator. Cells were seeded in a 96-well microtiter tissue culture plate and allowed to adhere overnight, and then treated with 2 μl anthranilate for 24 hours at various concentrations. Control cells were treated with 2 μl DMSO. To measure cell viability, 10 μl of water soluble tetrazolium reagent (EZ-CyTox, Daeil Lab Service) was added to each well and incubated at 37 °C for 1 hour. Absorbances were measured using a iMark Microplate Absorbance Reader (Bio-Rad) at a wavelength of 450 nm.

### Slime production assay

Slime production by *S. aureus* was assayed on Congo red agar plates (BHI medium containing 3.6% sucrose, 0.8 g/L Congo red, and 1% agar), as previously described^40^. Aliquots (3 μl) of overnight culture dropped on plates and incubated at 37 °C for 24 hours. Since slime is stained by Congo red, slime production by *S. aureus* appears dark brown around colonies on Congo red agar plates.

### Statistical analysis

The student’s *t*-test (two-sample assuming equal variances) in MS Office Excel (Microsoft) was used to determine the significances of differences. *P*-values of less than 0.05 were considered significant. At least, all experiments were repeated twice in triplicate.

## Results

### Anthranilate inhibits biofilm formation by a wide range of bacteria

**Figure 1:**
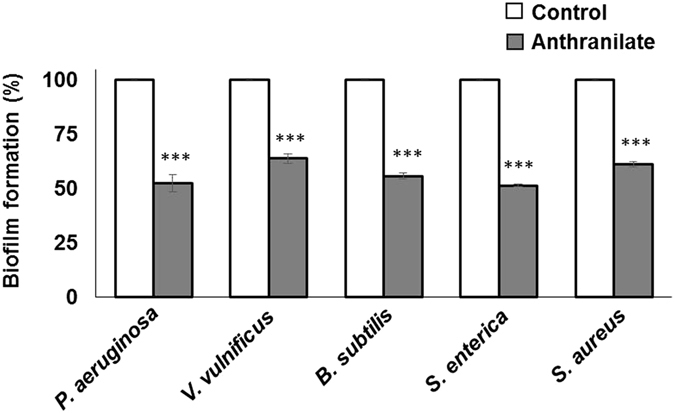
Inhibition of biofilm formation by anthranilate Static biofilm analysis was carried out with various bacteria. Biofilms were formed for 12–16 hours in a static biofilm system in the presence of 0.1 mM anthranilate. Since anthranilate was added to media from 1 M stock made up in dimethyl sulfoxide (DMSO), the same amount of DMSO was added to controls. Biofilm amounts were quantified by crystal violet staining as described in Materials and Methods and are presented as percentages of controls. ***p < 0.005.

For this study, we selected five bacterial species, that is, *P. aeruginosa*, *V. vulnificus*, *B. subtilis*, *S. enterica* serovar Typhimurium, and *S. aureus*, because these strains represent a variety of bacteria in terms of their genetic classification, whether or not they produce anthranilates, type of motility, and their importance (Table S2). We investigated the effect of anthranilate on biofilm formation in a static biofilm formation system. Biofilm formations of all bacterial species were significantly inhibited by 36.2% to 48.6% (Fig. 1). We note that in our previous study^23^, anthranilate was demonstrated to increase the initial attachment of *P. aeruginosa*, although it eventually decreased the biofilm formation. In the previous study, citrate and casamino acids were used for carbon source of culture medium, but in this study, we used glycerol medium in which the biofilm formation by *P. aeruginosa* was inhibited by anthranilate throughout growth.

**Figure 2:**
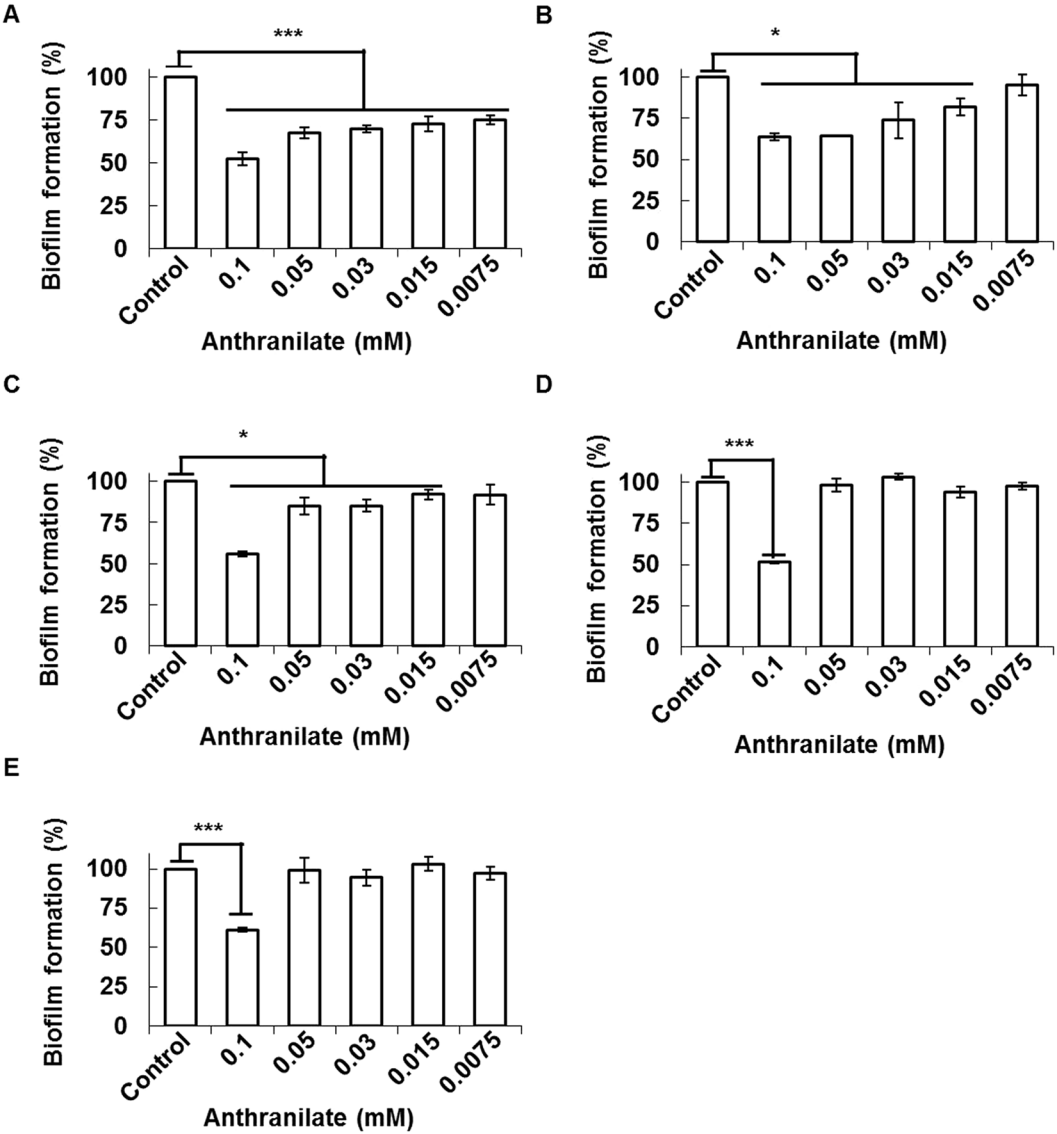
Effective biofilm-inhibitory concentration of anthranilate *P. aeruginosa* (**A**), *V. vulnificus* (**B**), *B. subtilis* (**C**), *S. enterica* (**D**), and *S. aureus* (**E**) were treated with different concentrations of anthranilate and biofilm formations were quantified by static biofilm analysis. Results are presented as percentages of the control. *p < 0.05; ***p < 0.005.

**Figure 3:**
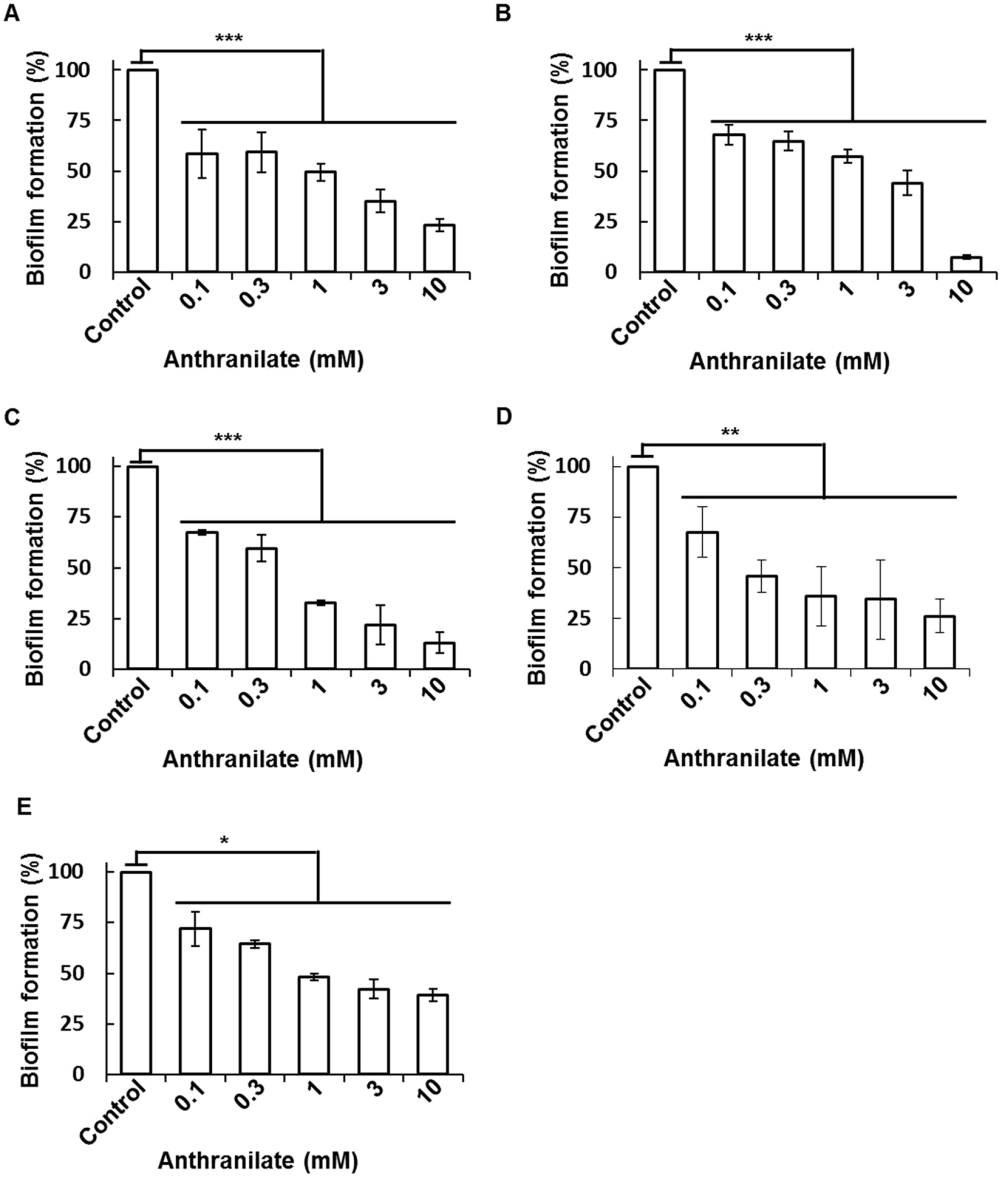
Strong inhibition of biofilms by high concentration of anthranilate *P. aeruginosa* (**A**), *V. vulnificus* (**B**), *B. subtilis* (**C**), *S. enterica* (**D**), and *S. aureus* (**E**) were treated with high concentration of anthranilate and biofilm formations were quantified by static biofilm analysis. Results are presented as percentages of the control. *p < 0.05; **p < 0.01; ***p < 0.005.

When we examined the minimum effective concentration of anthranilate on the biofilm formation, *P. aeruginosa* showed the highest sensitivity to anthranilate (lower than 7.5 μM) (Fig. 2). The minimum effective concentrations for other bacteria were as follows; *V. vulnificus* (15 μM), *B. subtilis* (15 μM), *S. enterica* (100 μM), and *S. aureus* (100 μM) (Fig. 2). Furthermore, we investigated biofilm inhibition at high concentration of anthranilate and the results showed that higher concentrations of anthranilate had greater inhibitory effects (Fig. 3).

**Figure 4:**
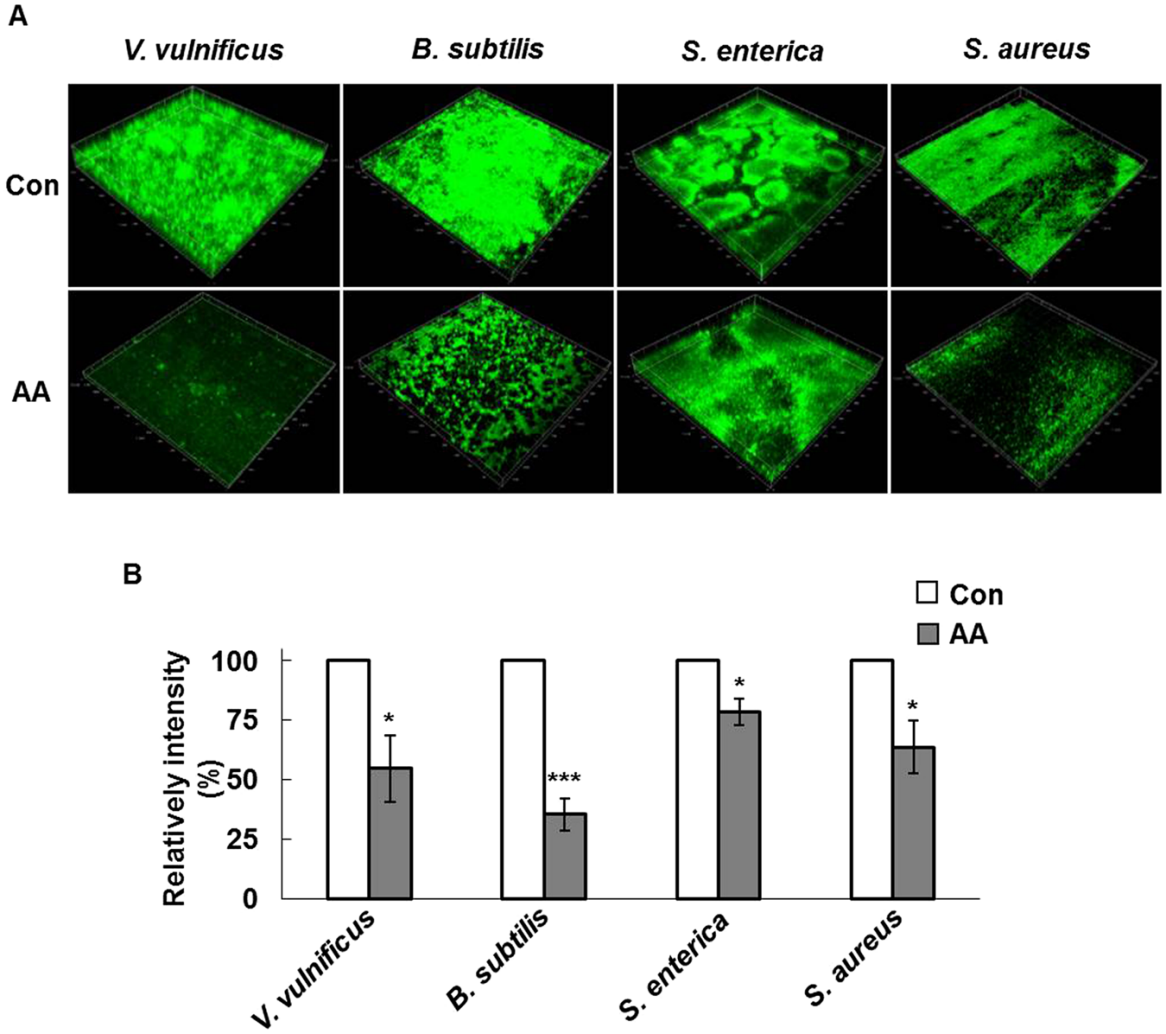
Biofilm inhibition by anthranilate in the presence of shear All strains were grown in flow cell chambers in the presence of 0.1 mM anthranilate for 3–5 days to form biofilms. *V. vulnificus*, *B. subtilis* and *S. enterica* biofilms were stained with 0.1% SYTO 9 for 15 minutes before microscopy, but *S. aureus* biofilms were detected directly because *S. aureus* harbors a GFP-expressing plasmid (pAH13). All biofilms were imaged by CLSM (**A**). Fluorescence intensities were quantified and graphed (**B**). Con, control; AA, 0.1 mM anthranilate. *p < 0.05; ***p < 0.005.

Anthranilate has been reported to inhibit biofilm formation by *P. aeruginosa* in a flow cell system^23^. Similarly, the biofilm inhibition by anthranilate was tested in the flow cell system for other four bacterial species and anthranilate was able to prevent biofilm formation by all bacterial strains in the flow cell system (Fig. 4). Taken together, we conclude that biofilm inhibition by anthranilate is common in a variety of bacteria.

### Anthranilate can disrupt preformed biofilms and induce detachment

**Figure 5:**
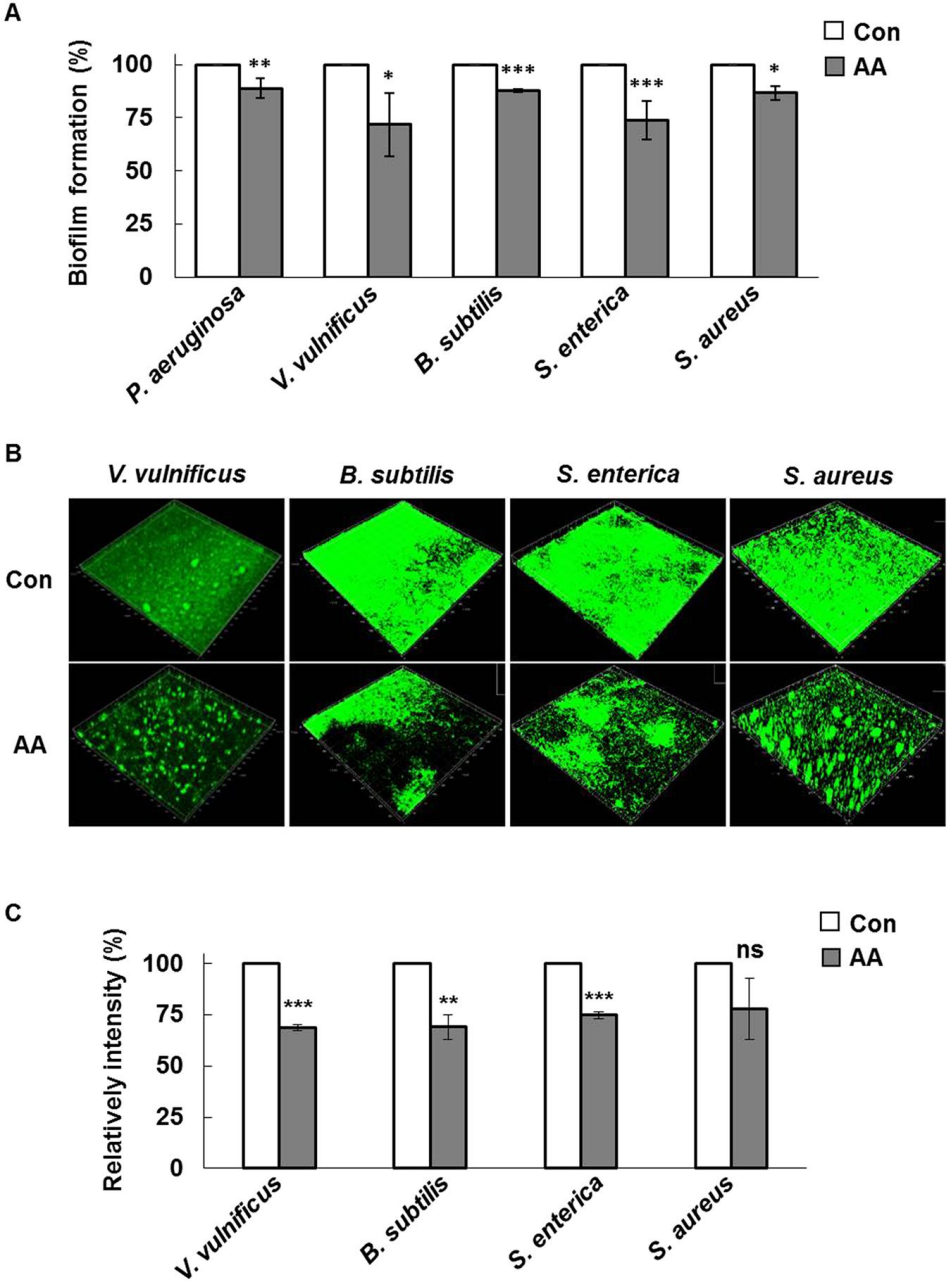
Detachment of preformed biofilms by anthranilate (**A**), bacterial biofilms were formed in the absence of anthranilate for 12 hours in the static biofilm system and then 0.1 mM anthranilate was added. After 12 hours of incubation, residual biofilms were quantified by crystal violet staining. (**B**) Biofilms were formed in the absence of anthranilate for 2–3 days in the flow cell system, and then treated with 0.1 mM anthranilate for 24 hours. A preformed biofilm was incubated without anthranilate under the same conditions as a control. All biofilms were imaged by CLSM (**B**) and fluorescence intensities were quantified (**C**). Con, control; AA, 0.1 mM anthranilate. *p < 0.05; **p < 0.01; ***p < 0.005; ns, no significant difference (*p* > 0.05).

Anthranilate has been reported to induce the detachment of preformed *P. aeruginosa* biofilms^23^. We first investigated biofilm detachment with other bacteria in the static biofilm system. Biofilms were preformed in the absence of anthranilate for 2 days and then anthranilate was applied for 12 hours. Crystal violet staining showed that biofilm masses of *V. vulnificus*, *B. subtilis*, *S. enterica*, and *S. aureus* were significantly decreased by anthranilate treatment (Fig. 5A).

The biofilm-detaching effect of anthranilate appeared weak in the static biofilm system. However, biofilm detachment is generally facilitated by shear force in the presence of flow. Therefore, we investigated biofilm detachment in the flow cell system. Preformed biofilm masses of *V. vulnificus*, *B. subtilis*, and *S. enterica* were significantly reduced by anthranilate treatment (Fig. 5B,C). Since preformed biofilms are generally firmly adherent to surfaces, this reduction in the biofilm mass by anthranilate treatment can be attributed to the biofilm detachment.

Interestingly, the mass of *S. aureus* preformed biofilm was not significantly reduced by anthranilate (Fig. 5C), although they tended to decrease. This result implies that the biofilm-detaching effect of anthranilate is not effective in *S. aureus*. The reason the ineffectiveness of anthranilate in *S. aureus* is discussed below.

### Anthranilate reduces intracellular c-di-GMP levels

**Figure 6:**
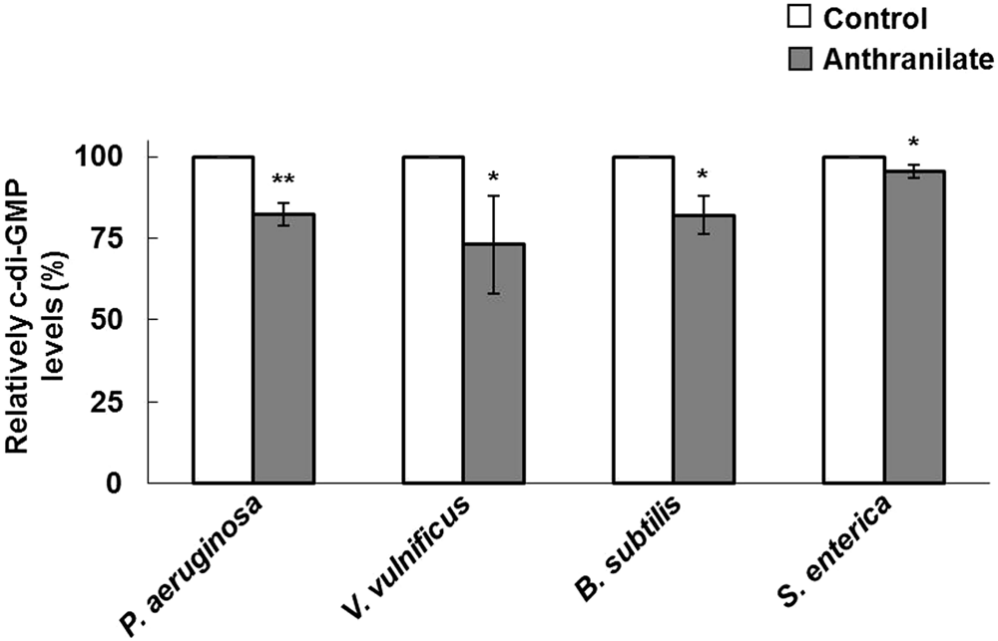
Reduction of intracellular c-di-GMP by anthranilate Intracellular c-di-GMP levels in various bacteria were measured after treatment with 0.1 mM anthranilate using a Thiazole orange (TO)-based fluorescence assay. Results are presented as percentages of the control. *p < 0.05; **p < 0.01.

There are several distinct fashions in biofilm detachment. Biofilm dispersion is an active detachment process triggered by several specific biological signals, the most important of which is a decrease in intracellular c-di-GMP (3′,5′-cyclic diguanylate) level^41^. Briefly, this decrease facilitates biofilm dispersion and a planktonic existence, whereas an increase in intracellular c-di-GMP facilitates biofilm formation and a sessile existence^42^. This c-di-GMP-mediated regulation of biofilms has been reported in a variety of microorganisms, and notably, anthranilate has been found to decrease intracellular c-di-GMP levels in *P. aeruginosa* ^23^. Accordingly, we investigated whether anthranilate affects biofilm formation by modulating intracellular c-di-GMP levels of *V. vulnificus*, *B. subtilis*, and *S. enterica*; intracellular c-di-GMP levels were assessed using a thiazole orange (TO)-based fluorescence assay (TO produces fluorescence when bound to c-di-GMP). This method was validated by comparing result with that of a *cdrA-lacZ* reporter-based measurement of c-di-GMP, which has been used in *P. aeruginosa* ^23^ (Fig. S1). In *P. aeruginosa*, intracellular c-di-GMP levels were reduced by 18% in the presence of anthranilate (Fig. 6, Fig. S1) and this reduction resulted in the dispersion of *P. aeruginosa* biofilms^23^. In *V. vulnificus*, *B. subtilis*, and *S. enterica*, anthranilate significantly reduced intracellular levels of c-di-GMP by 27%, 18%, and 5%, respectively (Fig. 6), which are comparable with that observed for *P. aeruginosa*. Thus, our results suggest that anthranilate induces biofilm dispersion by reducing intracellular c-di-GMP levels in *P. aeruginosa*, *V. vulnificus*, *B. subtilis*, and *S. enterica*.

Intracellular c-di-GMP levels were not measured in *S. aureus*, because intracellular c-di-GMP signaling does not exist in this species, despite the fact that *S. aureus* has a conserved GGDEF domain protein^43, 44^.

### Anthranilate enhances swimming and swarming motilities

**Figure 7:**
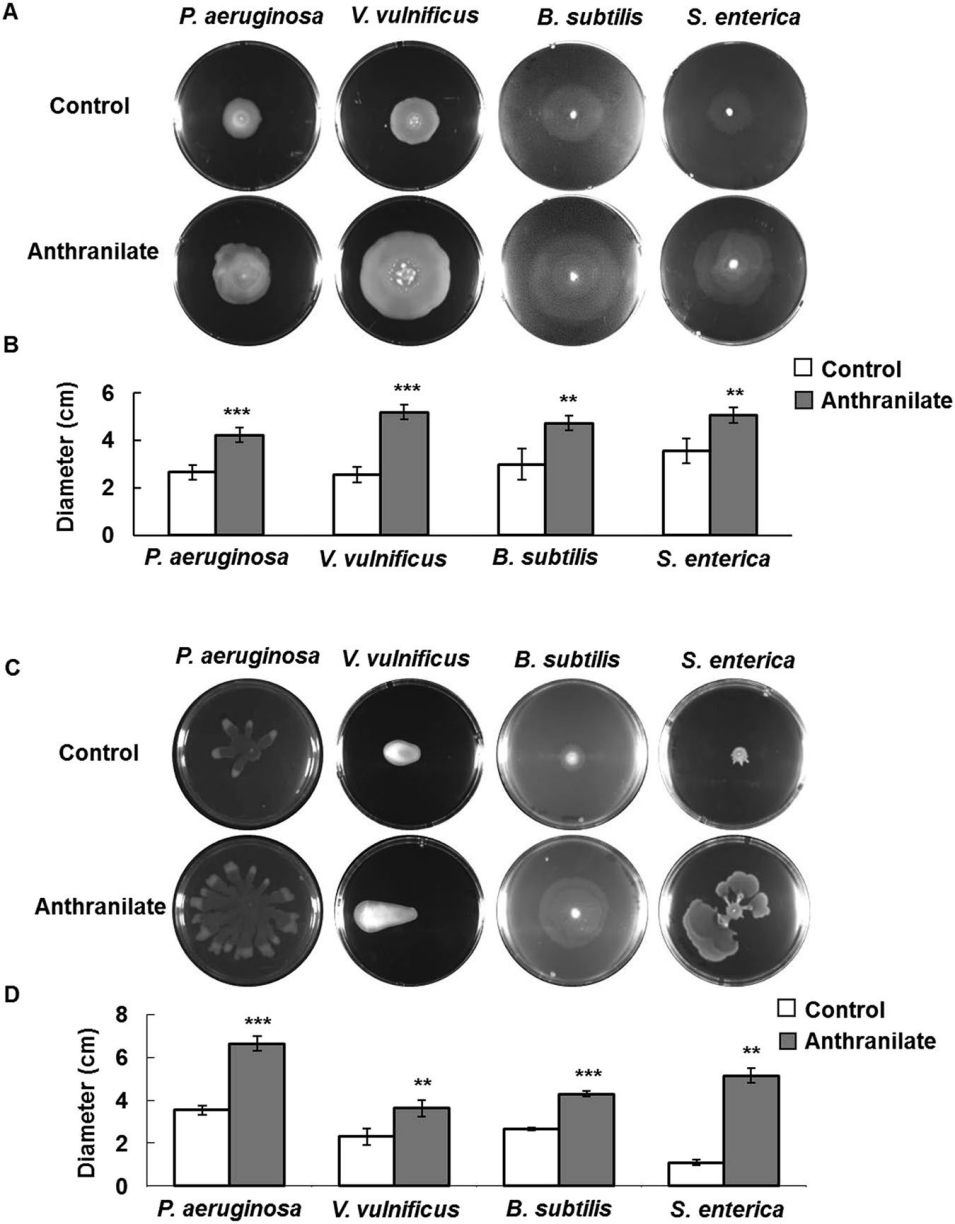
Effect of anthranilate on bacterial motility Swimming motilities of the bacterial strains were examined on semi-solid agar plates in the presence of 0.1 mM anthranilate (**A**). Diameters of swimming zones were graphed (**B**). Swarming motilities were also measured on solid agar plate containing 0.1 mM anthranilate (**C**) and diameters of swarming zones were graphed (**D**). **p < 0.01; ***p < 0.005.

Bacterial motility is regarded as a phenotype that should be repressed for irreversible attachment to surface at early stage of biofilm formation, but should be induced for biofilm dispersion^41^. Therefore, enhanced motility can accelerate biofilm dispersion^45–48^. We found anthranilate significantly enhanced the swimming and swarming motilities of *P. aeruginosa*, *V. vulnificus*, *B. subtilis*, and *S. enterica* (Fig. 7). These results are consistent with our biofilm dispersion results and indicate anthranilate facilitates biofilm dispersion by promoting swimming and swarming motilities and by decreasing intracellular c-di-GMP levels.

Swimming and swarming are flagella-dependent motilities whereas twitching is pilus-dependent. Interestingly, the twitching motilities of *P. aeruginosa*, *V. vulnificus*, *B. subtilis*, and *S. enterica* were not affected by anthranilate (Fig. S2), which suggests anthranilate targets flagellar function.

### Biofilm formation by *S. aureus* is inhibited by anthranilate in a different manner

Because *S. aureus* is a non-flagellated bacterium, we expected anthranilate would not affect its motility. *S. aureus* has a different type of motility, that is, colony spreading. In fact, anthranilate had no effect on the spreading motility of *S. aureus* (Fig. S3), which also reconfirmed anthranilate affects only flagella-dependent motilities. If so, the *S. aureus* biofilm reduction by anthranilate should have other reasons, because *S. aureus* does not have c-di-GMP signaling.

**Figure 8:**
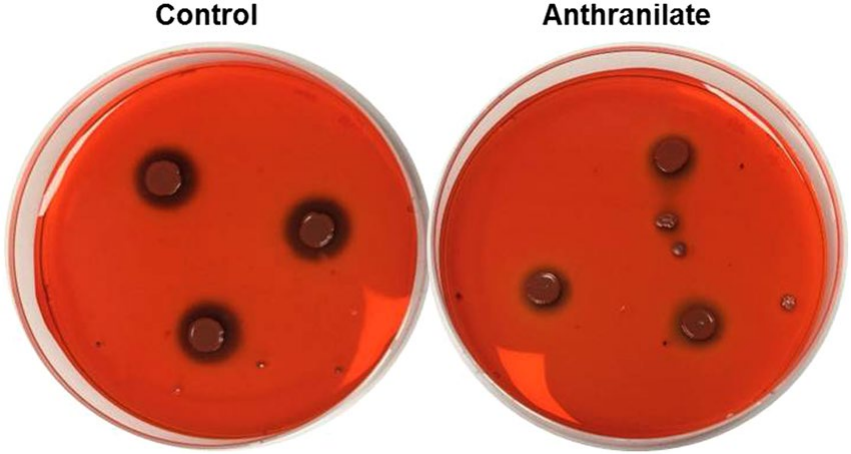
Inhibition of slime production by *S. aureus* by anthranilate Slime production by *S. aureus* was assessed by Congo red staining. *S. aureus* overnight culture (3 μl) was inoculated on a CRA plate containing 0.1 mM anthranilate and incubated at 37 °C for 24 hours. Slime production was visualized as a dark red around colonies.

In general, the EPS production is very important for biofilm maturation and in the case of *S. aureus*, staphylococcal slime is associated with biofilm formation as a major EPS^44^. When we tested slime production by *S. aureus*, we found it was significantly reduced by anthranilate treatment (Fig. 8). This result suggests that in *S. aureus*, anthranilate inhibits the biofilm maturation by interfering with the EPS production, rather than induces the biofilm dispersion by affecting c-di-GMP signaling or motility. This suggestion explains why the biofilm-detaching effect of anthranilate was not effective in *S. aureus* (Fig. 5C). Thus, it appears anthranilate inhibits bacterial biofilm formation in several multiple ways, that is, by affecting c-di-GMP signaling or motility to induce biofilm dispersion (in *P. aeruginosa*, *V. vulnificus*, *B. subtilis*, and *S. enterica*), or by suppressing EPS production to repress biofilm maturation (in *S. aureus*).

### Anthranilate is more effective at inhibiting biofilms than _L_-tryptophan

**Figure 9:**
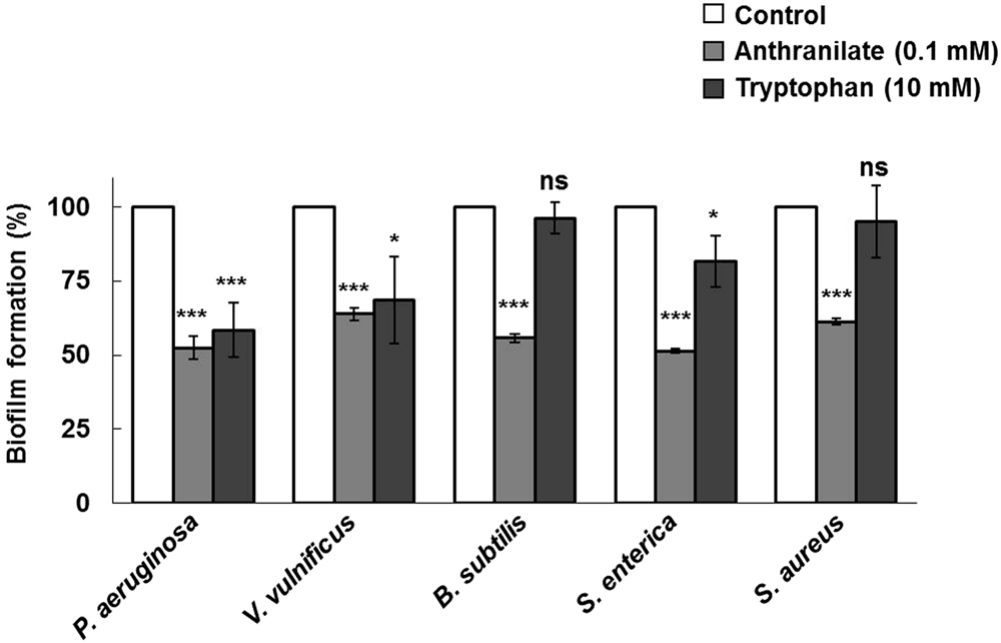
Comparison of the anti-biofilm effects of anthranilate and L-tryptophan The anti-biofilm effects of L-tryptophan (10 mM) and anthranilate (0.1 mM) were compared by static biofilm analysis. Results are presented as percentages of controls. *p < 0.05; ***p < 0.005; ns, no significant difference (*p* > 0.05).

L-Tryptophan at concentrations >1.6 mM has been shown to repress biofilm formation by *P. aeruginosa* ATCC 27853 strain by 50% and to enhance swimming motility^16^. Since this tryptophan effect on the biofilm is similar to the anthranilate effect observed in this study, we compared the anti-biofilm effects of anthranilate and tryptophan. In our comparison with *P. aeruginosa* PAO1 strain, L-tryptophan showed an inhibitory effect at 10 mM, whereas anthranilate exhibited a similar effect at 0.1 mM (Fig. 9). Similarly, about 10 mM L-tryptophan was required for half-level inhibition of the *V. vulnificus* biofilm, whereas anthranilate had a similar inhibitory effect at 0.1 mM (Fig. 9). L-Tryptophan showed a slight inhibitory effect on *S. enterica* biofilm, and no effect on *B. subtilis* and *S. aureus* biofilms even at 10 mM, whereas anthranilate at 0.1 mM had significant effects on all bacterial strains examined (Fig. 9). This result suggests that anthranilate is more effective in biofilm inhibition and has broader application range.

Even if it has a good effect, it should be non-toxic to develop into an anti-biofilm substance. So, we examined the toxicity of anthranilate to human cells. When HepG2 cells (an immortalized human hepatocyte cell line) were exposed to anthranilate for 12 hours, anthranilate did not cause cell death or lysis even at a concentration of 10 mM (Fig. S4). This result demonstrates that anthranilate has no direct cytotoxic effect.

## Discussion

Our results show the following: 1) anthranilate exerts biofilm-inhibiting effects on a wide range of bacteria; 2) in *P. aeruginosa*, *V. vulnificus*, *B. subtilis*, and *S. enterica*, anthranilate reduces intracellular c-di-GMP levels and enhances swarming and swimming motilities to induce biofilm dispersion; 3) in *S. aureus*, anthranilate reduces slime production to inhibit biofilm formation. In our previous work, we found anthranilate can disrupt the biofilm structure of *P. aeruginosa* and finally making a flat biofilm^23^. In this study, we further investigated the effects of anthranilate on biofilm formation by various bacterial species and found that anthranilate had antibiofilm effects on all bacterial species tested. Since the strains used in this study represented various bacterial physiology and characteristics (Table S2), these results indicate anthranilate has antibiofilm effects on a wide range of bacterial species.

Then, how does anthranilate deteriorate the biofilm? Developmental cycle of biofilm includes multiple stages; initial attachment of planktonic cells, microcolony formation, biofilm maturation in EPS matrix, and biofilm detachment^49, 50^, and thus, biofilm formation can be prevented by inhibiting cell attachment at early stage or interfering with EPS production at middle stage, or inducing biofilm detachment at late stage^51–53^. Anthranilate was found to have two effects on *P. aeruginosa*, *V. vulnificus*, *B. subtilis*, and *S. enterica*, that is, it reduced intracellular c-di-GMP levels and enhanced motility, which are related to biofilm detachment at late stage. Biofilm detachment is the most general term to indicate the reduction of biofilm mass and can be classified as: 1) sloughing, 2) erosion, and 3) seeding dispersal^41^. Sloughing and erosion are passive processes induced by shear forces, whereas dispersal is active process by biological activity^41^. We suggest anthranilate probably induces dispersal, because of its effects on intracellular levels of c-di-GMP and motility.

Studies have demonstrated negative relation between motility and biofilm formation, as the inhibition of motility has been shown to enhance biofilm formation by a wide range of microorganisms^54^. In *B. subtilis*, the *eps* operon that synthesizes EPS also encodes a bifunctional protein EpsE that inhibits flagella motility by blocking flagella rotor during biofilm formation^55, 56^. In *P. aeruginosa* and *V. cholera*, motility inhibition caused by repressing flagella gene expression is achieved by intracellular c-di-GMP up-regulation during biofilm formation^57, 58^. In *E. coli*, both the activation of capsular biosynthesis and suppression of flagella biogenesis occur by Rcs phosphorelay^59, 60^. According to these studies, the inhibition and enhancement of motility are requirements of biofilm formation and dispersal, respectively, and our observations that anthranilate inhibited *P. aeruginosa*, *V. vulnificus*, *B. subtilis*, and *S. enterica* biofilm formation by enhancing swarming and swimming motilities are consistent with the findings of these previous studies. Furthermore, it would seem anthranilate only enhances a flagella-mediated motility, as non-flagellar motilities, such as the twitching of flagellate bacteria and the spreading of *S. aureus*, a non-flagellate bacterium were not affected by anthranilate (Fig. S2, Fig. S3).

However, anthranilate seems to have effects other than the induction of dispersion. We previously reported anthranilate disrupted *P. aeruginosa* biofilm formation by reducing intracellular c-di-GMP levels and modulating EPS expression. In that study, we have found that the anthranilate modulates the expressions of the synthetic genes for Psl, Pel, and alginate which are major components of EPS^23^. This finding indicates anthranilate can influence biofilm maturation as well as dispersion. Furthermore, in this study, anthranilate reduced slime production by *S. aureus* (Fig. 8), which is probably responsible for the anti-biofilm effect of anthranilate on *S. aureus*, because this bacterium does not possess a c-di-GMP signaling system and its motility was unaffected by anthranilate.

Anthranilate is a natural product, has a simple molecular structure, is cheap and widely used in industry. When we compared the effectivenesses of L-tryptophan and anthranilate on the biofilm inhibition, anthranilate was found to be more potent than L-tryptophan (Fig. 9). Our cytotoxicity study showed anthranilate had no cytotoxic effect (Fig. S4). Anthranilate is naturally produced in man and animals by the kynurenine pathway, which is the major route for the metabolism of L-tryptophan^61, 62^. So, we suggest that anthranilate is a promising substance for the development of anti-biofilm agent.

## Electronic supplementary material

Supplemental dataset
