# Supplementary material for: Anti-biofilm effects of anthranilate on a broad range of bacteria
Source: Sci Rep. 2017 Aug 17;7:8604. doi: 10.1038/s41598-017-06540-1 (PMC5561115; doi:10.1038/s41598-017-06540-1)
Supplement: Supplementary file 1 — Supplemental dataset [file 41598_2017_6540_MOESM1_ESM.doc]

**Anti-biofilm effect of anthranilate on broad range of bacteria**

**Xi-Hui Li, Soo-Kyoung Kim, and Joon-Hee Lee***

**Department of Pharmacy, College of Pharmacy, Pusan National University, Busan, 609-735, South Korea**

**Table S1.** Bacterial strains and plasmids used in this study

| Name | Description^a^ | Reference |
| --- | --- | --- |
| Strains | | |
| *P. aeruginosa* PAO1 | A wild type strain of *P. aeruginosa* | 1 |
| *V. vulnificus* M06-24/O | A wild type strain of *V. vulnificus* | 2 |
| *S. aureus* AH1121 | *S. aureus* RN4220 strain harboring pAH13 | 3 |
| *S. enterica* SL1344 | A type of *S. enterica* serovar Typhimurium | Lab. collection |
| *B. subtilis* ATCC6051 | A wild type strain of *B. subtilis* | Lab. collection |
| *E.coli* DH5α | *supE44 ΔlacU169 (φ lacZΔM15) hsdR17*  *recA1 endA1 gyrA96 thi-1 relA1* | Lab. collection |
| Plasmids |  |  |
| pAH13 | GFP-expression plasmid, Erm^r^ | 3 |
| pSKcdrA | *cdrA-lacZ* fusion in pQF50, Ap^r^ | 4 |

^a^ Ap^r^, ampicillin and carbenicillin resistance; Erm^r^, erythromycin resistence

**Table S2. Description of bacterial strains used in this study**

| Bacterial species | Gram (+)/(-) | flagellum | c-di-GMP signaling | importance | | Tryptophan metabolite |
| --- | --- | --- | --- | --- | --- | --- |
| *P. aeruginosa* | **-** | + | + | Human pathogen | Anthranilate | |
| *V. vulnificus* | **-** | + | + | Human pathogen | Indole | |
| *S. aureus* | **+** | - | - | Human pathogen | Anthranilate | |
| *S. enterica* | **-** | + | + | Human pathogen | Indole | |
| *B. subtilis* | **+** | + | + | Industrial strain | Anthranilate | |

**
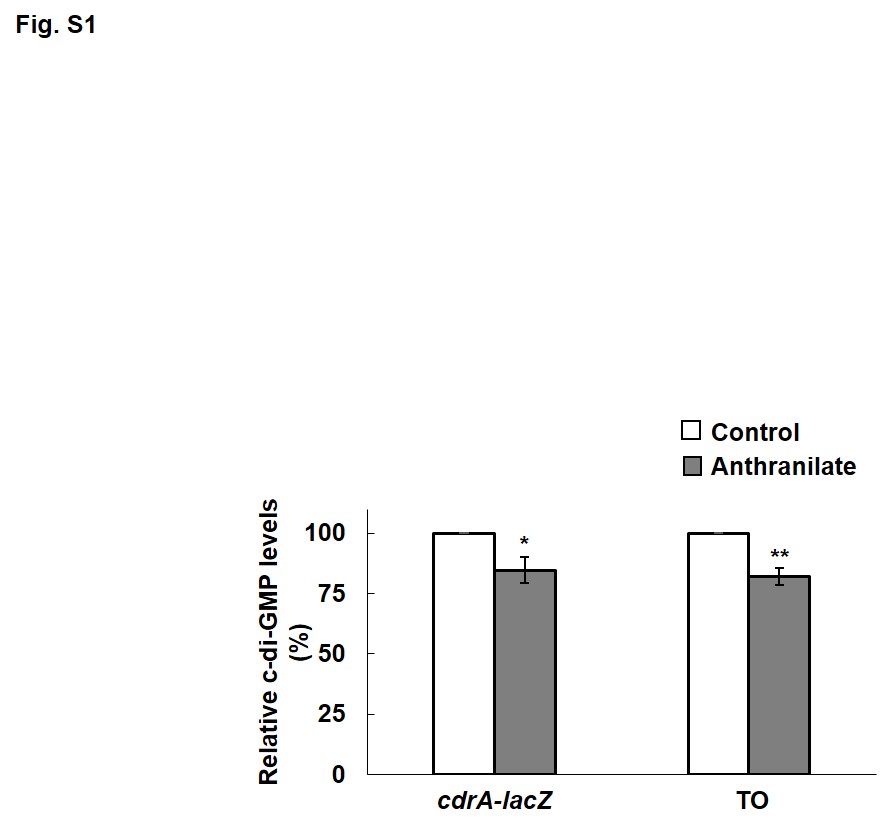
**

**Fig. S1. Comparison of two methods for measuring intracellular c-di-GMP levels in *P. aeruginosa*** The intracellular c-di-GMP levels of *P. aeruginosa* were measured by two different methods in the same condition for comparison. A, *cdrA-lacZ* reporter plasmid (pSKcdrA) was introduced to *P. aeruginosa* by transformation and β-galactosidase activity was measured as described previously^5^. *cdrA* is up- or down-regulated in response to intracellular levels of c-di-GMP. B, the c-di-GMP level was measured by Thiazole orange (TO)-based fluorescence assay. c-di-GMP levels are presented as percentages of controls. Anthranilate was added at a concentration of 0.1 mM. *, *p* < 0.05; **, *p* < 0.01

**
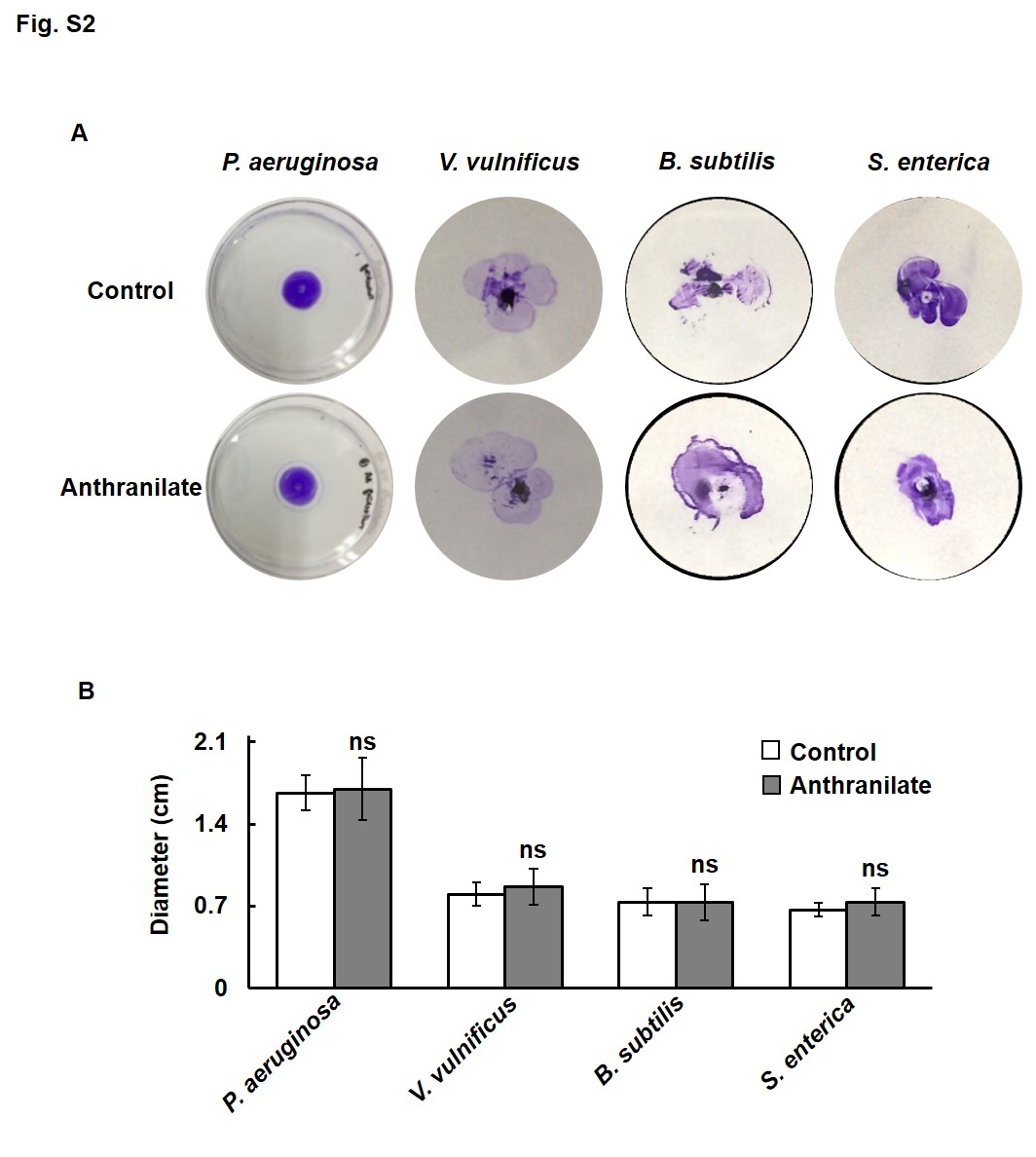
**

**Fig. S2. Effects of anthranilate on twitching motility** Twitching motilities of *P. aeruginosa*, *V. vulnificus*, *B. subtilis*, and *S. enterica* were assayed as described in Materials and Methods. Anthranilate was added at 0.1 mM. The twitching zones were visualized by staining (A) and diameters were graphed (B). ns, no significant difference (*p* > 0.05).

**
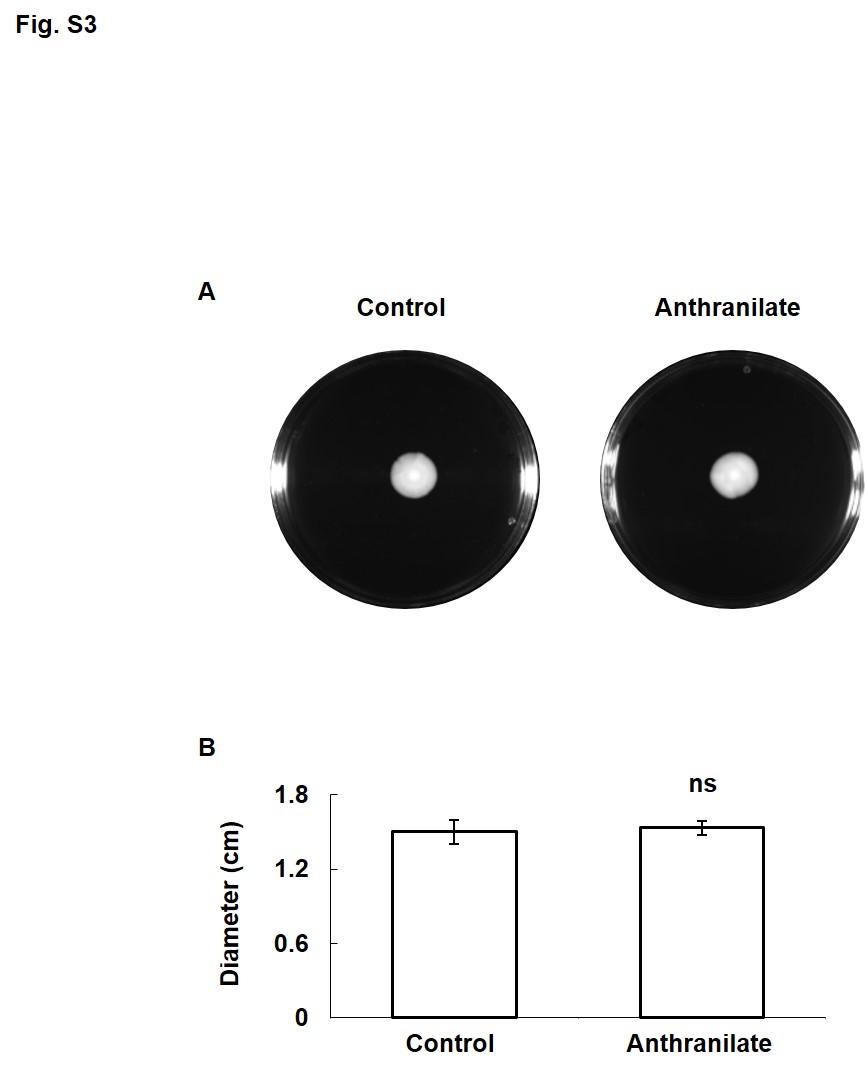
**

**Fig. S3. Effect of anthranilate on *S. aureus* spreading motility** The spreading motility of *S. aureus* was measured on 0.3% agar plates containing 0.1 mM anthranilate (A) and diameters were graphed (B).

**
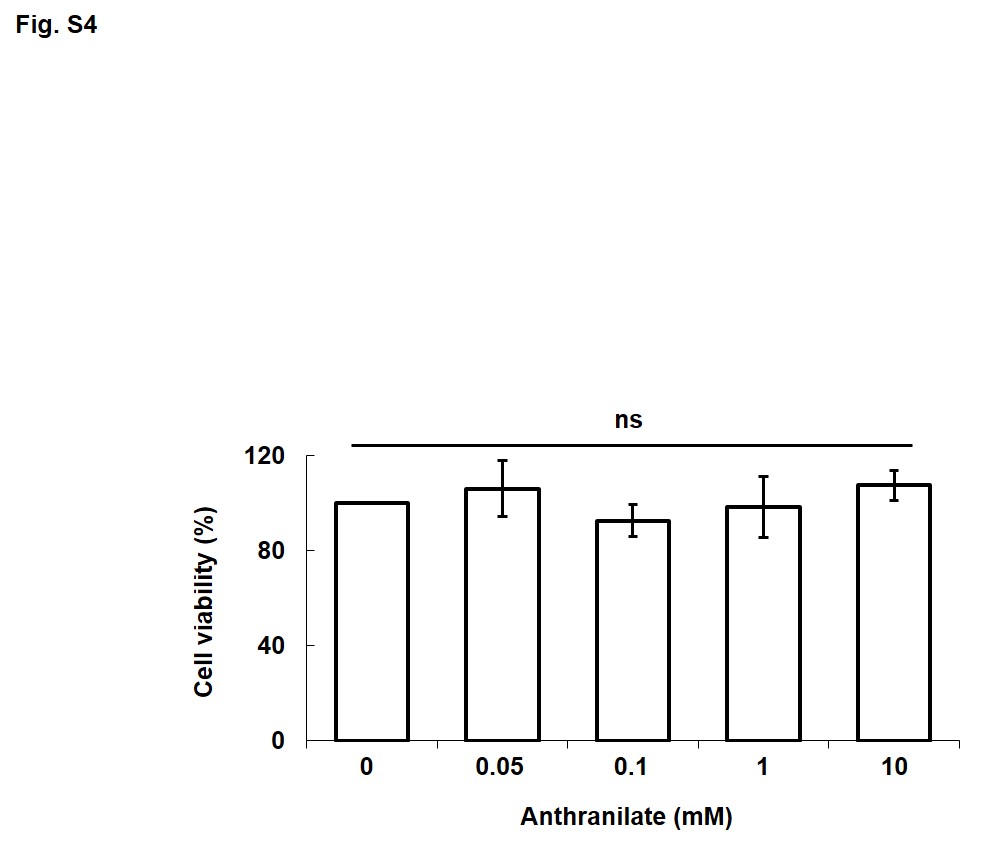
**

**Fig. S4. Cytotoxicity of anthranilate on HepG2 cells** When HepG2 cells (an immortalized human hepatocyte cell line) were posed to anthranilate for 12 hours at 37°C, no cytotoxic effect was observed. Cell viabilities were assayed by adding tetrazolium reagent and measuring absorbance at 450 nM. Viabilities are presented as percentages of non-treated controls. ns, no significant difference (*p* > 0.05).

**References for supplementary data**

1 Pearson, J. P., Pesci, E. C. & Iglewski, B. H. Roles of Pseudomonas aeruginosa las and rhl quorum-sensing systems in control of elastase and rhamnolipid biosynthesis genes. *Journal of bacteriology* **179**, 5756-5767 (1997).

2 Roh, J.-B. *et al.* Transcriptional Regulatory Cascade for Elastase Production in Vibrio vulnificus LuxO ACTIVATES luxT EXPRESSION AND LuxT REPRESSES smcR EXPRESSION. *Journal of Biological Chemistry* **281**, 34775-34784 (2006).

3 Malone, C. L. *et al.* Fluorescent reporters for Staphylococcus aureus. *Journal of microbiological methods* **77**, 251-260 (2009).

4 Kim, S.-K., Park, H.-Y. & Lee, J.-H. Anthranilate deteriorates the structure of Pseudomonas aeruginosa biofilms and antagonizes the biofilm-enhancing indole effect. *Applied and environmental microbiology* **81**, 2328-2338 (2015).

5 Kim, S. K., Park, H. Y. & Lee, J. H. Anthranilate deteriorates the structure of Pseudomonas aeruginosa biofilms and antagonizes the biofilm-enhancing indole effect. *Applied and environmental microbiology* **81**, 2328-2338, doi:10.1128/AEM.03551-14 (2015).
